# Evaluating the impact of personalized rehabilitation nursing management in the perioperative nursing of patients with intracranial aneurysm: A protocol for systematic review and meta-analysis

**DOI:** 10.1097/MD.0000000000029121

**Published:** 2022-07-15

**Authors:** Qiong Wang, Wei Qiu

**Affiliations:** a The First People’s Hospital of Jiangxia District, Wuhan City (Union Jiangnan Hospital Huazhong University of Science and Technology), Wuhan, Hubei, China.

**Keywords:** intracranial aneurysm, meta, perioperative nursing, personalized rehabilitation nursing

## Abstract

**Background:**

Patients who suffer from aneurysmal subarachnoid hemorrhage continue to die at a high rate, despite large geographical and risk factor-related variability in death rates. Hemorrhagic stroke has a greater death rate than ischemic stroke, which is often permanent, although patients who survive have less impairment than those who do not. Because of this, nurses must offer appropriate nursing care throughout the perioperative period for patients suffering from this prevalent neurological condition. Patients with intracranial aneurysms (IA) will be the subjects of this research, which will be carried out to thoroughly investigate the effect of tailored rehabilitative nurse treatment throughout the perioperative period.

**Methods:**

The influence of customized rehabilitative nurse management in the perioperative nursing of patients with IA has been studied in both randomized controlled trials and observation studies, according to the authors. From the time of the database’s creation until February 2022, information about studies will be gathered from 3 Chinese databases (Wanfang, China National Knowledge Infrastructure, and Chinese BioMedical database) and 4 English databases (PubMed, EMBASE, Cochrane Library, and CINAHL database). After the data extraction and risk of bias evaluation of the included studies are completed, the data synthesis will be carried out using the RevMan 5.3 program. Egger’s regression test and funnel plots will be used to determine whether or not there is any publication bias. The *I^2^* statistics will be used to determine the degree of heterogeneity. A sensitivity analysis will be conducted to determine the robustness as well as stability of our results and conclusions.

**Results:**

This research is anticipated to offer high-quality evidence of tailored rehabilitative nurse treatment in the perioperative nursing of patients with IA.

**Conclusion:**

Results of this research will synthesize the current data to determine whether or not tailored rehabilitation nursing treatment may enhance the surgical recovery of patients with IA.

## 1. Introduction

With a high possibility of occurring, an intracranial aneurysm (IA) is considered among the most frequent disorders in clinical neurosurgery.^[1]^ It is produced by subarachnoid hemorrhage, which is brought about by aberrant bulging on the wall of the intracranial artery.^[1]^ An estimated 3% of the adult population has unruptured IAs.^[2]^ Not every IA has a propensity to rupture, and the yearly incidence of IA rupture is roughly 1%.^[3]^ A number of relevant investigations have shown that IA are on the rise globally, with a tendency toward a steady increase year by year.^[4]^ They are the third most common kind of cerebrovascular illness, behind only cerebral thrombosis and hypertensive intracerebral hemorrhage.^[4]^ Stroke-related morbidities as well as mortalities in the United States are exacerbated by IA rupture, which is a prevalent etiologic component of subarachnoid hemorrhage.^[5,6]^ Even though IA is typically asymptomatic, a tiny number of cases may rupture, resulting in life-threatening health implications.^[7]^ Before an IA rupture occurs, the primary motivation for IA therapy has been to avoid future IA rupture, which might result in subarachnoid hemorrhage and the accompanying morbidity and mortality concerns.^[7–9]^

Perioperative nursing of patients undergoing surgical therapy has long been recognized as being required in clinical practice.^[10]^ Perioperative nursing is now the focus of nursing research, yet it is also a tough topic to address. Some clinical investigations have shown that improving the effectiveness of nursing measures in the operation room may minimize the length of time patients with IA spend in the hospital and the number of adverse responses they experience.^[11]^ Full nursing focused on risk prevention included a comprehensive risk event assessment for patients with IA who were treated surgically, as well as targeted intervention strategies for patients who had a risk event.^[12]^ With the continual growth in people’s level of life comes an increase in the demand for medical standards and services, which has resulted in the normal nursing mode being unable to satisfy the needs of the vast majority of patients. Rehabilitation nursing that is tailored to the specific condition and needs of each patient is a new nursing model that uses clinical evidence gathered through literature consultation to formulate a scientific and reasonable nursing plan that is then adjusted in real time according to the effectiveness of nursing measures in order to achieve the best nursing outcome. Patients with IA will be included in this research, which will be done to comprehensively investigate the effect of tailored rehabilitative nursing care in the perioperative nursing of these patients.

## 2. Methods

### 2.1. Study registration

This study has been registered in the Open Science Framework (https://osf.io/) with number of 10.17605/OSF.IO/S4RJG. This protocol is reported based on the Preferred Reporting Items for Systematic Reviews and Meta-Analysis Protocol statement guidelines.^[13]^

### 2.2. Inclusion criteria for study selection

#### 2.2.1. Types of studies.

There will be randomized controlled trials as well as observational studies investigating the efficacy of customized rehabilitative nursing management in the perioperative nursing of patients with IA.

#### 2.2.2. Types of participants.

Participants will be of any age, gender, or ethnicity diagnosed with IA using digital subtraction angiography, craniocerebral computed tomography, and magnetic resonance imaging.

#### 2.2.3. Types of interventions.

Individually tailored rehabilitative nursing care should be provided to the experimental group. The control group should be employed given regular nurse treatment throughout the perioperative phase.

#### 2.2.4. Types of outcome measures.

The Glasgow Coma Scale score after being awake, the length of postoperative hospital stays, nurse satisfaction, the frequency of complications, and the incidence of infection are all outcome measures.

### 2.3. Search methods for the identification of studies

#### 2.3.1. Electronic searches.

From the time of the database’s creation until February 2022, information about studies will be gathered from 3 Chinese databases (Wanfang, China National Knowledge Infrastructure, and Chinese BioMedical database) and 4 English databases (PubMed, EMBASE, Cochrane Library, and CINAHL database). An algorithm will be used to get information from the database using a mix of free words and medical topic headings such as “intracranial aneurysm,” “personalized rehabilitation,” “perioperative nursing,” “randomized controlled trials,” and other terms.

#### 2.3.2. Searching other resources.

We will undertake a manual search of all retrieved papers’ references, look for unpublished trials and abstracts presented to major international conferences, and contact study authors and manufacturers to see if they are aware of any further studies that have been conducted.

### 2.4. Data collection and analysis

#### 2.4.1. Selection of studies.

As a consequence of the search approach, we found a large number of potential studies, which 2 authors would independently evaluate for inclusion. We will attempt to address any differences via conversation, and if necessary, we will seek the advice of a third author.

#### 2.4.2. Data extraction and management.

Using a predefined data extraction form, 2 authors will extract the basic information and outcome information from the included studies. This information will include, but not be limited to, the included trials, the first author’s name, the publication date, the sample size, the intervention methods used in the experimental group and control group, the age and gender of the participants, the outcome indicators, adverse events, and a risk of bias assessment. We will attempt to address any differences via conversation, and if necessary, we will seek the advice of a third author.

#### 2.4.3. Assessment of risk of bias in included studies.

According to the researchers, the Cochrane risk of bias tool will be used to analyze randomized controlled trials, and the Newcastle-Ottawa Quality Assessment Scale will be employed in examining observational studies. The quality of each research will be evaluated by 2 independent writers who will work together on the project. We will try to address any differences via conversation, and if necessary, we will seek the advice of a third author.

#### 2.4.4. Data synthesis and analysis.

The *I^2^* statistic value will be used to investigate the heterogeneity of the data. If there is no indication of high heterogeneity (*I^2^* <50%), a fixed effect model will be utilized; otherwise, a random effect model will be used to analyze the data. Mean difference with 95% confidence interval (CI) will be used to show the treatment impact for continuous data, and relative risk with 95% CI will be used to indicate the treatment effect for dichotomous data, according to the study. To evaluate publication bias, funnel plots and Egger’s regression test will be used. For all analyses, the level of statistical significance will be fixed at *P* < .05.

#### 2.4.5. Sensitivity analysis.

To assess the robustness and stability of pooled data, the methodological quality, and the presence of missing data, a sensitivity analysis will be carried out.

## 3. Discussion

This meta-analysis aims to determine the effect of tailored rehabilitative nurse care in the perioperative nursing of patients with vascular access disease. The outcomes of this research will give evidence as to whether tailored rehabilitative nurse care will result in promising advantages in the perioperative nursing of patients with IA, which is currently unknown. The findings of this systematic review and meta-analysis will be useful information for doctors in making judgments in clinical practice, as well as for health policymakers in making decisions about health policy. Results of this research will synthesize the current data to determine whether or not tailored rehabilitation nursing treatment may enhance the surgical recovery of patients with IA.

## Author contributions

WQ and QW were responsible for study conception, data acquisition, data interpretation, and design. All authors have read and approved the final manuscript.
